# Early Career Outcomes of Embedded Research Fellows: An Analysis of the Health System Impact Fellowship Program

**DOI:** 10.34172/ijhpm.2023.7333

**Published:** 2023-02-22

**Authors:** Bahar Kasaai, Erin Thompson, Richard H. Glazier, Meghan McMahon

**Affiliations:** ^1^CIHR Institute of Health Services and Policy Research, Toronto, ON, Canada.; ^2^ICES (Institute for Clinical Evaluative Sciences), Toronto, ON, Canada.; ^3^MAP Centre for Urban Health Solutions, St. Michael’s Hospital, Toronto, ON, Canada.; ^4^Department of Family and Community Medicine, University of Toronto, Toronto, ON, Canada.; ^5^Institute of Health Policy, Management and Evaluation, University of Toronto, Toronto, ON, Canada.

**Keywords:** Health Services, Policy Research, Health Workforce, Career Development, Learning Healthcare System

## Abstract

**Background:** This descriptive study reports the early career outcomes of postdoctoral fellows who completed a novel embedded fellowship training program, the Canadian Institutes of Health Research (CIHR) Health System Impact (HSI) Fellowship. The program was designed to support impact-oriented career paths of doctoral graduates, build research capacity within health system organizations, and help to advance learning health systems in Canada.

**Methods:** Employment of fellowship alumni upon completion of the program were tracked using internet searches of publicly accessible online sources and complemented with program survey data.

**Results:** Descriptive analyses show that all 87 eligible alumni included in the study are currently employed (100% of 87), with 92% employed in Canada. Their employment spans several sectors, including in academic (37%), public (29%), healthcare delivery (17%), and private (14%) sectors. Altogether, 32% of alumni held hybrid roles with an affiliation in academia and another sector. The most common position types were senior scientist (42%), professorships (18%), and director, manager or administrator roles (12%). Program reporting data indicate that these employment outcomes are generally consistent with the group’s career aspirations reported at the start of the fellowship program, and that the program receives high ratings from fellows in the extent it is believed to support their career preparedness and readiness (4.49 out of 5).

**Conclusion:** We find that HSI Fellow alumni are employed mostly in research-related roles in a range of sectors including, but not limited to academia, that they positively perceive the program’s success in elevating their career readiness and potential to make an impact – suggesting that the program may help equip fellows with the skills, readiness and networks for a broad array of employment sectors and roles. The findings are a promising signal of the demand for research talent and the growing capacity for learning health systems in Canada.

## Background

**Key Messages**

** Implications for policy makers**

- An examination of the career pathways of Canada’s first major investment in embedded trainees, the Health System Impact (HSI) Fellowship program, reinforces the need for PhD and postdoctoral training and curricula to evolve and modernize to prepare trainees for diverse career pathways, bolster preparedness and skills to make an impact in those careers, and to prepare them for leadership roles in learning health systems (LHSs).
- The study offers considerations for future attention and policy as Canada works to advance LHSs, including building the human capital needed to support high-performing LHSs, fostering sustainable embedded research and LHS-related career paths, developing data systems to track and understand PhD career pathways and using the data to inform PhD and PhD alumni training improvements.
- Other policy implications and actions could include developing an early career embedded research program and supporting the work of the pan-Canadian Training Modernization Task Force to incorporate the enriched core competencies and experiential learning opportunities within doctoral curriculum across the country.

** Implications for the public**

Two shifts in the healthcare and academic sectors are mobilizing attention and action to modernize health services and policy research (HSPR) training programs: evolving health system needs for human resources and a relative decline in academic positions. Faced with rising costs, post-pandemic challenges, and systemic problems with access, equity, and outcomes, health system organizations (HSOs) in Canada and abroad are striving to build capacity for rapid learning and improvement. This study examines the employment outcomes of doctoral graduates of a novel embedded research fellowship program, which aimed to support diversified, impactful career paths and build research capacity within HSOs. Our findings of the high employability of program alumni in diverse HSO is viewed as a promising signal of the demand for research talent, the growing learning health systems (LHSs), and the positive influence training programs may have to help bolster the size of the research workforce in Canada.

Changing health system needs and a relative decline in academic tenure-track positions are mobilizing attention and action to modernize health services and policy research (HSPR) training programs to ensure they prepare graduates to maximize their contribution within and beyond the academy, particularly within health system organizations (HSOs) that are committed to rapid learning and improvement. Faced with rising costs and systemic problems with access, equity, quality and outcomes, HSOs in Canada and abroad are increasingly striving to build capacity for rapid learning and improvement and move towards a learning health system (LHS) model.^1-3^ This model, first proposed by the National Academy of Medicine as a framework for transforming care delivery and outcomes,^4^ positions research as directly aligned to the operational priorities of the healthcare system to help organizations learn in real time, and adapt and improve their services, policies, products, processes and outcomes.^3,5,6^ To foster the LHS model and support HSOs to use data and evidence to inform decision-making, investments are needed to build the required human capital (research capacity) within health systems to drive agile, rapid cycle innovation, implementation and evaluation.^7-11^ This evolution towards LHSs creates opportunities for HSPR doctoral graduates to apply their research skills to analyze real-world challenges, engage with health system leaders, test innovations, develop and implement practical solutions, and pursue impactful careers in a range of sectors, including sectors within and beyond the traditional academic setting.^11-13^ At the same time, the academic sector has experienced a decline in the number of tenure-track professor positions relative to the number of PhD graduates and increase in the number of PhD graduates employed outside academia.^12,14^ PhD career outcome data indicate that, depending on the study, 60%-80% of PhD graduates now enter careers in roles and sectors other than tenure-track university professor.^12,15^ A key question is whether doctoral and postdoctoral training has evolved to prepare trainees to maximize their contribution and potential for impact in LHSs and other sectors and roles that are increasingly outside of the academy. Several recent studies and initiatives suggest that incorporating opportunities for experiential learning and enhancing the suite of traditional research competencies in which doctoral students receive training could improve trainees’ career preparedness and impact potential for careers in diverse sectors and roles.^10,11,15-17^ The present study considers this question by examining the career pathways of alumni of a novel embedded research graduate training program in Canada.

###  Training Modernization and the Canadian Institutes of Health Research’s Health System Impact Fellowship

 In 2015, Canada’s HSPR community identified the potential significance of these healthcare and academic sector trends and prioritized the need to modernize HSPR training to optimize the impact potential of the future HSPR workforce. A pan-Canadian Training Modernization Strategy was produced outlining a five-point plan for collective action across universities, HSOs and research funders.^18^ The strategy identified the need for HSPR doctoral training to evolve to include professional competencies (eg, leadership, change management, communication) in addition to research skills, opportunities for experiential learning within HSOs, and better career outcome tracking to understand graduates’ diverse and evolving employment pathways. The enriched core competency framework for HSPR was a key outcome of the Training Modernization Strategy.^16^ The competency framework outlined a suite of 10 competencies deemed essential to training doctoral students for impact and contribution in a diverse range of sectors and roles. Notably, the framework was informed with input from health system leaders and health system employer organizations, along with researchers and other stakeholders, and features several professional competencies identified as critical by employers (eg, leadership, change management, project management, dialogue and negotiation) but that are underemphasized or not yet reflected in most PhD training programs.

 In response to this pan-Canadian Training Modernization strategy, the Canadian Institutes of Health Research-Institute of Health Services and Policy Research (CIHR-IHSPR) ngaged with partners across the country to develop an embedded fellowship program in 2017 called the Health System Impact (HSI) Fellowship.^19^ The HSI Fellowship aims to (1) support diverse career paths of PhD trainees and postdoctoral fellows, (2) elevate fellows’ readiness to contribute and make an impact in a diverse range of health sectors and roles, (3) enhance embedded research capacity within HSOs, and (4) contribute to advancing LHSs in Canada. To achieve these aims, the program provides fellows with an embedded, experiential training opportunity within an HSO where they lead a project designed to address an impact goal (ie, high priority issue) of the organization. In addition, fellows receive co-mentorship from a health system leader within the organization and an academic at a Canadian university; are supported to develop their enriched core competencies (eg, leadership, change management) with a professional development training allowance, a self-assessment tool, and training offerings organized by the program team; and join a national cohort training program comprising all the fellows and their respective mentors, which fosters opportunities for networking, collaboration, and shared learning. Postdoctoral fellows receive a two-year stipend to dedicate the majority of their time (at least 70%) focusing on their impact-oriented program of work, and the remaining time (up to 30%) is protected for academic research. Fellows’ dual roles within an HSO and university and their protected time for academic research are intended to help them maintain and develop their research skills and networks, and to apply these assets to their embedded research with the HSO. The program’s distinctive features and their contribution to fellows’ competency development and impact within HSOs have been described elsewhere.^16,20-25^

###  Study Aim and Contribution to the Literature

 A critical gap in the training modernization and embedded fellowship literature is evidence on the career outcomes of PhD graduates and, in particular, embedded researchers. To help address this evidence gap, and to understand whether the HSI Fellowship attains its program objectives to support diverse career pathways and enhanced career preparedness, this descriptive study investigates the employment trends of the first graduating cohorts of the program.

 The current study contributes to the relatively scant evidence on the HSPR workforce and PhD career pathways in Canada and abroad. It builds on a previous study of the pan-Canadian employment trends of graduates of HSPR doctoral programs over a 20-year period,^12^ which found that HSPR PhD employment trends changed over time: today’s graduates are more likely to enter careers in a wider variety of sectors and roles and are less likely to be employed in academia than previous graduates. Another study of a postdoctoral health policy training program in Georgia (USA) found that 84% of alumni were employed in a variety of sectors upon graduation, including government agencies, non-profit sectors, and the academy.^26^ Otherwise, the literature in doctoral graduate career tracking studies is generally sparse and not specific to HSPR or embedded research.^27-29^ The limited available evidence generally reports high employability of alumni and illustrate that post-PhD career pathways extend well beyond the traditional academic setting. This raises questions as to whether and how training programs should evolve their curriculum to improve the trainees’ preparedness to adapt and apply skills in the real-world context, and the evidence needs to understand HSPR trainee career pathways, particularly at the PhD level.

 Since its inception in 2017, the HSI Fellowship program has supported four cohorts and more than 200 fellows who have been embedded in over 100 HSOs across Canada. This is a considerable investment in embedded research capacity and LHSs in the country, yet the post-fellowship career pathways of program alumni and their perspectives on career readiness have not yet been examined. The HSI Fellowship program is now at a stage of maturity that allows for analysis of fellowship-to-career transitions among alumni that have completed the program. This study addresses a recognized evidence gap pertaining to the career pathways of PhD graduates, and embedded researchers in particular, and aims to provide an initial descriptive profile of the employment outcomes of the first three cohorts of HSI postdoctoral fellows, including the sectors, organizations, and roles in which alumni are employed. This study also reports on fellows’ perspectives of career readiness and the program’s contribution to their readiness.

## Methods

###  Study Sample and Eligibility

 The eligibility criteria includes postdoctoral fellows who were awarded their HSI Fellowship during the 2017 to 2019 study period and who completed their fellowship. Exclusion criteria included fellows for whom employment data could not be reliably extracted (n = 4), fellows still enrolled in the HSI Fellowship at the time of analysis due to program extensions or other factors (n = 8), and fellows who renewed their award for another term and thus participated twice in the program (n = 8). The resulting study sample includes 87 HSI Fellow alumni who commenced their two-year fellowship in 2017, 2018, or 2019. Therefore, the duration of time post-fellowship for career development differs across cohorts.

###  Career Tracking Methods

 The current study builds on previously published methods for tracking the career pathways of HSPR doctoral graduates,^12^ which were informed and adapted from the work of the University of Toronto’s 10 000 PhDs Project and other studies assessing doctoral graduates’ employment outcomes that used publicly accessible online sources for employment tracking.^30-33^

 The career information of the HSI Fellow alumni in the present study was predominantly extracted from publicly available information from social media (particularly the employment-oriented social media platform, LinkedIn (n = 79), and in a few cases, other sources (n = 3) such as ResearchGate and Twitter). The extracted data were confirmed with website searches of the employer organizations and/or data from start-of-grant survey data (when available).

 Informed by the HSPR PhD career tracking study^12^ and the University of Toronto’s 10 000 PhDs Project,^33^ a standardized career tracking template and corresponding codebook with definitions of the variables was developed and used for data extraction. The template included an established HSPR-specific taxonomy for employment sectors, and role types^16^ and the taxonomy included seven key sectors: academic, public, healthcare delivery, healthcare delivery research (ie, research department within a hospital), private, not-for-profit, and other. The first and last names of the HSI Fellow alumni were the primary variables used to identify and track them. For each alumnus, the current primary employment position(s) were extracted from social media and documented, including their specific job title (eg, senior scientist in a governmental organization and adjunct assistant professor at a university), and the name, geographical location, and sector of the organization. Additional variables included: fellow name, name of HSO where fellow was embedded, current academic affiliation (yes or no) and, if applicable, the type of academic affiliation (eg, professor-adjunct/status, research associate/assistant, instructor/lecturer). No further demographic information was consistently available or gathered from social media sources.

###  Fellowship Program Reporting Data

 Social media-extracted career data were complemented with the program’s start-of- and end-of-fellowship reporting data, which included an analysis of the fellows’ self-reported sex, career aspirations (at the start of their fellowship), and satisfaction levels with the extent to which the HSI Fellowship program and their supervisors supported their career preparedness (at the end of the fellowship). The fellows’ sex variable was extracted as a self-reported variable from their *Canadian Common CV* questionnaire submitted at the time of Fellowship application (response options included: female, male, prefer not to answer). At the outset of the fellowship, Fellows were asked in a baseline survey to “identify their short term (fellowship), medium term (2-5 years) and long term (5+ years) career goals.” This allowed for comparison of initial career goals at the start of the fellowship (data available from 76 of the same fellows) with post-fellowship career outcomes (n = 87). Further, at 12 and 24 months into the Fellowship, fellows were asked to rate, based on a 5-point Likert type scale (1 = not at all, 5 = significantly): (1) the extent to which the fellowship elevated their career readiness and preparedness to make an impact, and (2) their satisfaction of their supervisors’ interests in and support of their career prospects (n = 56 and 49 respondents, respectively, of the same study sample).

###  Descriptive Analyses

 Unless otherwise specified, the unit of analysis in this study is the fellow (fellows for whom employment data were available). All employment data were collected and analyzed between September and December 2021, triangulated with program reporting data, and reported in aggregate form with identifiable information removed for publication. Descriptive analyses were used to summarize alumni characteristics, fellowship-to-career outcomes, and perspectives on the program’s contribution to their career readiness. Sex-based differences in employment outcomes and self-rating scores were determined by the chi-squared test and two-sample *t* test for means, respectively.

 All analyses were conducted, and tables created, in Excel. Beyond the mandatory program reporting requirements described above, no contact was made with fellows, their fellowship supervisors, universities, or employers regarding their employment.

## Results

###  Study Sample Characteristics and Fellows’ Self-reported Reflections on Their Career Pursuits

 Of the 91 postdoctoral fellows who had completed the fellowship by the time of the study and were therefore eligible for inclusion in the career tracking study, 87 could be successfully tracked through social media (96% tracking rate). Table 1 illustrates a descriptive summary of the 87 alumni and their geographic distribution (top of table), and details on their HSI Fellowship HSOs (bottom of table), which were from the following HSI Fellowship program cohorts (where cohort year represents the start year of their two-year fellowship): 2017 (n = 45), 2018 (n = 21) and 2019 (n = 21). Overall, the fellows self-identified as predominately female across cohorts (76% of 87 total alumni).

**Table 1:** Study Sample Characteristics of Fellows and Their Health System Organization

	**2017 Cohort**	**2018 Cohort**	**2019 Cohort**	**Cohorts Combined**
Fellow characteristics (n = 87 total), and career profiles				
Funded postdoctoral fellows	52% (45)	24% (21)	24% (21)	100% (87)
Female fellows	80% (36)	62% (13)	81% (17)	76% (66)
Repeat fellows^a^		7	1	8
Geographic distribution of fellows^b^	West: 15% (13) Central: 33% (29) East: 3% (3)	West: 14% (12) Central: 9% (8) East: 1% (1)	West: 8% (7) Central: 16% (14) East: 0% (0)	West: 37% (32) Central: 59% (51) East: 5% (4)
HSO characteristics (n = 70 total), where fellows were embedded				
Unique organizations^a^	50% (35)	26% (18)	24% (17)	100% (70)
Public sector	24% (17)	17% (12)	7% (5)	49% (34)
Healthcare delivery	13% (9)	4% (3)	14% (10)	31% (22)
Private sector (for profit and not-for-profit)	13% (9)	4% (3)	3% (2)	20% (14)

Abbreviation: HSO, health system organization. ^a^ Several fellows and HSOs participated in more than one cohort. ^b^ Geographic regions were based on location of the HSO and defined as: West = British Columbia, Alberta, Saskatchewan, Manitoba, North Western Territories, Yukon Territory; Central = Ontario, Quebec; East = Nova Scotia, New Brunswick, Newfoundland and Prince Edward Islands.

Table 2 illustrates the fellows’ initial career aspirations (at the outset of the fellowship program) and their perception of the extent to which the fellowship program and their supervisors supported their career preparedness (at 12-months and 24-months into the fellowship). At the start of the fellowship, the majority of fellows reported interest in a researcher/scientist role (59%), either as an embedded position in a HSO (32%), in an undecided/ unspecified sector (14%), or as an independent researcher/faculty position in academia (13%). Several fellows aspired to careers as health system leaders (13%) or other positions such as knowledge brokers (5%). Almost a quarter of fellows (22%) indicated a career goal involving a hybrid role that would allow them to span academia and the broader health system.

**Table 2:** Fellows’ Initial Career Aspirations and Final Perception of the Fellowship’s Contribution in Elevating Career Readiness

	**2017 Cohort** **(n = 40)**	**2018 Cohort** **(n = 19)**	**2019 Cohort** **(n = 17)**	**Cohorts Combined ** **(n = 76)**	**% Female Fellows ** **(n = 54)**
Fellows’ baseline career aspirations (n = 76 total)					
Researcher, embedded in health system	18% (14)	8% (7)	4% (3)	32% (24)	63% (15)
Researcher, hybrid (health system + academia)	9% (7)	5% (4)	8% (6)	22% (17)	76% (13)
Researcher, unspecified/undecided setting	8% (6)	4% (3)	3% (2)	14% (11)	100%^a^(11)
Researcher/faculty position, academia	7% (5)	3% (2)	4% (3)	13% (10)	60% (6)
Health system leader, in health system	8% (6)	4% (3)	1% (1)	13% (10)	60% (6)
Other position (eg, manager, knowledge broker)	3% (2)	-	3% (2)	5% (4)	75% (3)
**Self-reported Item**				**12-Month Rating (n = 56)**	**24-Month Rating (n = 49)**
Fellows’ rating of the extent to which the fellowship and their supervisors’ support contributed to their career readiness (5-point Likert scale)					
Extent the Fellowship program elevated the fellows’ career readiness and preparedness to make an impact				4.27 (0.78)	4.49 (0.72)
Fellows’ satisfaction of their supervisors' interests in, and support for, their career pursuits				4.56 (0.74)	4.44 (0.97)

^a^Significant difference in career aspirations between male and female alumni (in the “researcher, unspecified/undecided” goal only), as per chi-square test (*P* < .05). No sex-based significant differences in self-reported items.

 HSI Fellowship reporting data indicate that fellows felt that the program elevated their career readiness and preparedness to make an impact (mean rating score of 4.49 out of 5), and that their supervisors supported their career pursuits (mean rating score of 4.44 out of 5).

 There were no significant sex differences between male and female career aspirations nor in fellows’ ratings of the program’s and their supervisors’ contributions to their career readiness (data not shown), except for more female alumni reporting an aspiration to be a researcher in an undecided/unspecific sector (Table 2, row three, *P* < .05).

###  Employment Characteristics

 Post-fellowship, among the 87 alumni whose employment could be tracked, Table 3 shows that the most common sector of employment was academic (37%). Within the academy (n = 32 alumni), half (50%) were assistant or associate professors, and 28% continued with additional postdoctoral training. Employment in the public sector was the next most common career path (29%), followed by the healthcare delivery research (17%) and private sector (14%) (Table 4). The majority of alumni resided and worked in Ontario (45%), followed by British Columbia (20%), Quebec (9%), and other provinces in Canada (10%), whereas 8% resided and worked abroad, including in the United States, the United Kingdom, and Europe.

**Table 3:** Primary Employment Sector and Location

**Employment Sector**			**Employment Location**		
**Sector**	**% Alumni (n = 87)**	**% Female (n = 66)**	**Province**	**% Alumni (n = 87)**	**% Female (n = 66)**
Academic	37% (32)	75% (24)	Ontario	45% (39)	79% (31)
Public	29% (25)	80% (20)	British Columbia	20% (17)	76% (13)
Healthcare delivery research	17% (15)	80% (12)	Quebec	9% (8)	38%^a^ (3)
Private, for-profit	8% (7)	57% (4)	Alberta	8% (7)	86% (6)
Private, not-for-profit	6% (5)	60% (3)	Other provinces^b^	10% (9)	89% (8)
Healthcare delivery	3% (3)	100% (3)	Other country	8% (7)	71% (5)

^a^ Significant difference between sexes (Quebec location only, row three), as per chi-square test (*P* < .01). ^b^ Other provinces include: Nova Scotia, Saskatchewan, Manitoba, New Brunswick.

**Table 4:** Type of Position Categorized to Employment Sector

**Type of Position**	**% Alumni (n = 87)**	**% Female (n = 66)**
Academic positions	**37% (32)**	**75% (24)**
Professor (assistant, associate)	18% (16)	69% (11)
Post-doctoral or research fellow	10% (9)	78% (7)
Director, manager or administration	3% (3)	100% (3)
Senior researcher/scientist (eg, epidemiologist)	5% (4)	75% (3)
Other sector positions	**63% (55)**	**76% (42)**
Senior researcher or scientist – *all other sectors*	37% (32)	78% (25)
Senior researcher or scientist – *public*	18% (16)	81% (13)
Senior researcher or scientist – *healthcare delivery Research*	11% (10)	70% (7)
Senior researcher or scientist – *private (for profit and not-for-profit)*	7% (6)	83% (5)
Director or manager – *not-for-profit, public, private, healthcare delivery *	9% (8)	75% (6)
Knowledge translation or mobilization expert – *public, healthcare delivery research *	6% (5)	80% (4)
CEO, co-founder and/or consultant – *private*	2% (2)	50% (1)
Postdoctoral Fellow – embedded in *healthcare delivery research, Public*	5% (4)	75% (3)
Other^a^	5% (4)	75% (3)
Employed by same HSO as HSI Fellowship^b^	**22% (19)**	**74% (14)**
Hybrid role – Professional affiliation with academia and another sector	**32% (28)**	**82% (23)**

Abbreviations: HSO, health system organization; HSI, Health System Impact; CEO, Chief Executive Officer. ^a^Other positions include clinician-scientist in *healthcare delivery research*, social worker in *healthcare delivery*, or senior program lead in *public* sector. ^b^Employed by the same HSO, where HSI Fellowship was completed/embedded.

Table 4 provides further detail about the types of employment positions held by HSI Fellowship alumni. The most common types of employment positions were senior researcher or scientist (37%), academic professor (18%), director or manager (9%), followed by knowledge translation/mobilization expert (7%), with coverage of these positions observed across almost all sectors. Regardless of sector of employment, the majority of alumni held research-related employment roles and 32% held a formal academic affiliation with a university (eg, as adjunct professor, lecturer, instructor) in addition to their employment in another sector (“hybrid role”). Nineteen alumni (22%) were hired by the same HSO in which they were embedded for their HSI Fellowship, including in the public sector (42%, n = 8 fellows), healthcare delivery research (37%, n = 7), not-for-profit (16%, n = 3) and healthcare delivery (5%, n = 1) sectors. There were no significant sex-based differences in alumni employment sectors or locations, with the exception that more males were employed in Quebec than females (*P* <.01).

 A comparison of the fellows’ initial career aspirations (at the start of the fellowship, Table 2) with their career outcomes (post-fellowship, Table 4) reveals general consistency between their career aspirations-to-outcomes at the aggregate program level, but less so at the individual level (ie, when the datasets were linked to the fellow; data not shown). For instance, at the aggregate (program cohort) level, 32% of fellows aspired to hold an embedded scientist position at the start of their fellowship and 37% of alumni held embedded scientist positions post-fellowship; however, at the individual level, 54% of alumni who specifically reported this goal attained it. Similarly, a hybrid role was an aspiration for 22% of alumni and an outcome for 32%, whereas at the individual level, 25% who specifically reported this goal attained it. An academic faculty position was an aspiration for 13% of respondent alumni and an outcome for 18%, whereas at the individual level, 40% of those who reported this goal attained it.

Box 1 illustrates the variety of job titles, occupations and organizations in which HSI Fellowship alumni are currently employed.

**Box 1. Illustrative Examples of Employment Organisation and Positions of Health System Impact Alumni**

Examples of the diverse types of *organizations* where the HSI alumni are employed include: provincial health authorities, federal health agencies, public health agencies, pharmaceutical or other private companies, academic teaching and community hospitals, their embedded research units, and other service delivery organizations, not-for-profits, universities, academic-affiliated research hubs, private companies founded by the fellows, and more.

Examples of *current employment roles* include: scientific/executive director, implementation scientist, senior scientist, senior methodologist, director of programs, biostatical lead, epidemiologist, clinical scientist, consultant, research and evaluation specialist, senior policy advisor/lead, senior policy analyst, knowledge translation expert, improvement consultant, entrepreneur/CEO, assistant/associate professor, Canada research chair, postdoctoral/research fellow, and more.

----------------

Abbreviations: HSI, Health System Impact; CEO, Chief Executive Officer.

## Discussion

 This descriptive study provides a first glimpse of the fellowship-to-career employment transitions of embedded research fellows that were part of the CIHR HSI Fellowship program, including the sectors, subsectors, occupations, and locations in which alumni currently work. The HSI Fellowship was designed to help bolster the career preparedness and impact potential of doctoral graduates by providing them with experiential learning, mentorship, professional development in an enriched set of core competencies, and national cohort networking opportunities, and also accelerate the evolution of LHSs by increasing research capacity directly within the health system. While this descriptive study cannot draw a causal link between fellows’ participation in the program and their post-fellowship employment success, the emerging picture is promising. The data reveal that, from the perspective of fellows at the end of their fellowship, the majority perceive the program greatly or significantly elevated their career readiness and preparedness to make an impact (mean rating of 4.49 on a 5-point Likert-type scale) and also report high levels of satisfaction with their supervisors’ support of their career pursuits (mean rating of 4.44 on a 5-point Likert-type scale).

 The study findings also reveal that HSI Fellows are highly employable upon completion of their fellowship training. Their career paths are diverse and span several sectors. All alumni whose career path could be tracked were employed (100% of 87), with 37% employed in the academy and the remaining 63% employed in the public, private (for profit and not-for-profit), and healthcare delivery sectors. Among the fellows continuing in the academy (n = 32 fellows or 37%), half (50% or n = 16) were in professor positions, whereas 26% (n = 9) continued onto postdoctoral positions. Almost a third of alumni (32%) held hybrid roles, primarily involving primary employment in the broader health system with an academic affiliation (31%). Most alumni, regardless of sector, were employed in research-related positions, which is a promising sign for the future of LHSs in Canada. Given previous findings that the inaugural cohort’s (professional and analytic) competencies enriched significantly over the course of the fellowship,^24^ it seems probable that the 63% employed outside of the academy bring research and professional skills, real-world embedded research experience, and access to a vast pan-Canadian network of emerging and established leaders to their employment positions. Equally, the 37% employed in the academy likely bring professional skills, experience and networks to their research, including insight into health system challenges, knowledge of how to design solution-oriented research questions, and ability to work in partnership with decision makers. The majority of fellows self-identified as female (76% of the sample), but sex-based differences in employment positions, sectors and locations were generally not significant.

 Analysis of alumni’s medium and long-term career aspirations at the start of their HSI Fellowship indicates that their post-fellowship employment outcomes are generally consistent with initial career aspirations when analyzed at the program cohort level, but less so when analyzed at the individual level (linked). For example, more fellows attained an academic position (often professorships) than initially planned (at both the individual and cohort-level). This could reflect the evolution of fellows’ career preferences that shift with experience and opportunity gained throughout their fellowship. It could reflect fellows’ networks and relationships within the academy. It could reflect demand from universities for researchers with the skillset and relationships that HSI Fellows hold (solution-oriented research, co-design and partnered research, working with decision makers, leadership). Or, it could reflect these and/or other factors, which future research could explore. The individual-level career goal-to-outcome alignment was poorest in the subset of fellows who aspired to be in health system leader roles (10% of fellows who specifically targeted this attained it), which may reflect alumni’s early career stage and the short length of time between their fellowship completion and this study’s career tracking. Overall, the individual-level disparity between goal-outcome may be due to a number of other reasons, including aspiring for a research role in one sector and attaining it in another (eg, an aspiring embedded scientist ended up in a hybrid role), minor coding biases, the labor market opportunities that were available or attractive upon fellowship completion, or other. Future research is needed to explore longer-term alignment between career goals and outcomes and the factors that influenced fellows’ post-fellowship employment path.

 Overall, this study corroborates previous studies that have also found that doctoral graduates are highly employable, and that employment is often outside of the academic sector.^12,32-34^ These findings were also observed in a recent Canadian study that focused specifically on HSPR doctoral graduate career trends over a 20-year time period.^12^ The Canadian study found that, over time, employment trends have changed and are increasingly beyond academic settings. HSPR graduates of the study’s most recent cohort (2015-2016) were less likely to be employed in academia than previous graduates: less than 30% of graduates from the 1996-1999 cohort were employed outside of academia whereas more than 60% of from the 2015-2016 cohort were. In addition, the research and key informant interviews that informed the creation of the pan-Canadian Strategy for Training Modernization^35^ found that HSPR PhD graduates entering in non-academic workplaces often reported feeling underprepared to effect change in their positions, and health system employers reported considerable differences between workplace cultures and essential skills in and outside academia.^16,35^ The HSI Fellowship design was informed by these broader PhD employment trends, the pan-Canadian Training Modernization Strategy, and the finding that doctoral graduate skills and the skills demanded in the non-academic labour market appear to be mismatched. It is therefore promising to observe in the present study that HSI Fellows equipped with experiential learning and embedded research expertise and training in the enriched core competencies were highly employable in careers within and beyond the academy, and that they perceived the fellowship to have elevated their career readiness and preparedness to make an impact.

 Based on the alumni’s career outcomes and their appraisal of the HSI Fellowship’s success in supporting their career readiness, we speculate that the program contributed to the alumni’s career prospects, readiness and employability directly and indirectly through its distinctive program design and features.^20-23,25,36^ First, the positive impacts on fellows’ employment transitions into careers in sectors beyond the traditional academic setting may be attributed in part to the program’s emphasis on (1) enriched core competency and professional development, intended to develop skillsets to achieve success beyond an academic environment^16,24^; (2) the dual mentorship from a health system leader within the organization and an academic at a Canadian university; and (3) a network of colleagues that span the country, currently comprised of 202 HSI fellows and alumni and their respective health system and academic supervisors, with a shared commitment to and expertise in evidence-informed health system improvement.^37^ This cohort and the network within has sparked multiple collaborations across fellows and mentors, including research studies, teams, and articles,^38-42^ a Network of Scholars program in Nova Scotia to advance LHSs,^9,43^ and leadership of the CIHR National Cohort Training Program.^44^ Second, similar to other training programs intended to provide experiential learning opportunities and diversify trainees’ potential career paths,^45^ a halo effect of the HSI Fellowship program’s emphasis on experiential learning likely augmented the fellows’ awareness of employment positions and sectors beyond academia through exposure to other career pathways in their networks and embedded environment. For example, through their embedded environments and participation in the national cohort that involves more than 100 distinct HSOs across Canada, HSI Fellows transition out of their fellowship with an understanding of career possibilities within and beyond academia and a network of potential employers that value research talent. Third, perhaps the most direct evidence of enhanced employability prospects is that upon Fellowship completion, 22% of alumni were hired by the same HSO where they were embedded. This illustrates the bidirectional benefits of the Fellowship on the fellows and the HSO.

 The HSI alumni reflected in this study are in the early stages of their post-fellowship careers. Future research that explores the contributions they make in their roles, their use of the professional skills and networks cultivated in their fellowship training, their satisfaction in their careers, and their subsequent career transitions and pathways will further illuminate the impacts of embedded training programs like the HSI Fellowship on employment outcomes.

 The early career outcomes of HSI Fellowship alumni are viewed as consistent with the program’s aims of enhancing the impact-potential and preparedness of doctoral holders in a wide array of employment sectors and increasing embedded research capacity within HSOs. The career outcomes reported in this and other PhD career trajectory studies that consistently find more graduates are increasingly entering science-related roles outside the university sector create an impetus and opportunity to reconceptualize notions of research impact and career success. Impact in the context of an embedded research program centers on real-world impact, including bringing relevant evidence to bear on complex decisions, building the capacity within HSOs to conduct and use evidence to make better informed decisions based on the best available science, and informing improvements in health policies, programs, services and outcomes.^6,25^

###  Study Limitations

 Our findings indicate that HSI Fellowship alumni are well positioned for promising careers within and beyond academia, that they perceive the Fellowship greatly contributed to their career readiness and preparedness to make an impact, and that their career outcomes closely aligned with their baseline career aspirations at the start of the fellowship at the aggregate level (less so at the individual level). Yet, this study is not without its limitations. The self-reporting data (career aspiration and their satisfaction with the Fellowship’s success in elevating their career readiness) were available for 49 to 76 of the 87 alumni (depending on the reporting item), possibly resulting in potential selection bias. Further, the absence of a control group of HSPR postdoctoral fellows sharing similar characteristics to the HSI Fellows but without exposure to the HSI Fellowship training means the study cannot fully attribute the career success of its alumni to the Fellowship and its program design elements. The program is highly competitive, and applications are adjudicated in a rigorous peer reviewed process by an expert panel using criteria that select for the academic and leadership potential of the applicant.^19^ Fellowship awardees are highly qualified and would likely otherwise, without the fellowship, have promising career paths, though it is not possible to discern the true counterfactual of what their employment outcomes would have been in the absence of the fellowship. Additionally, while sex as a demographic factor was available from self-reporting data and allowed for sex-based analyses in career outcomes (generally not significant), other demographic variables such as race or ethnicity were not available on social media nor in program reporting (privacy regulations). The employment outcomes of four fellows could not be ascertained using social media sources or end-of-fellowship report data, resulting in some missing data. Further, while social media enabled the tracking of near real-time career paths of alumni (allowing, for instance, the capturing of employment start date), there is a general lack of clarity on other important employment information, such as whether the positions were permanent or temporary (eg, on a contractual basis), part-time *vs.* full-time, tenure-track or not, salary levels, provision of benefits, career satisfaction, and employers’ perspectives on fellows’ skills, readiness and impact-potential. These are important factors to consider, as reported in a 2021 *Nature* survey.^46^ Additionally, it is challenging to confirm whether social media data are comprehensive and accurate.

 Although this study had access to end-of-fellowship reporting data that included a question on current employment, the study team determined that much of the employment information was outdated at the time of analysis, particularly for alumni from the 2017 and 2018 cohorts and who had more time in the workforce. Given the growing interest in PhD career outcomes, there is a need and opportunity to advance the methods and data for career tracking. To address these questions, future studies could use complementary methods and data sources, such as self-reported online surveys^47-49^ and semi-structured interviews.^50^

 Additionally, while the scope of the present study was to understand the early career paths of HSI alumni (within one month to three years of HSI Fellowship completion, depending on their cohort), in the future, an extended longitudinal study will shed important insight into career pathways and career progression of embedded researchers. It would also allow for investigation of whether the infusion of highly-skilled researchers within HSOs spurs the creation of new types of sustainable employment positions, such as Embedded Scientist or hybrid roles shared between a HSO and university, strengthens linkages between the academic and health system sectors, and increases system capacity for evidence-informed decision making and health system improvement.

## Conclusion

 Employment trends for HSI Fellow alumni appear bright. Upon completion of their embedded fellowship, alumni appraise the program’s success in elevating their career readiness and enter employment in a variety of sectors within and beyond academia. The finding that fellows are employed in the academic, public, private, healthcare delivery research and not-for-profit sectors in a variety of leadership and research-related roles is promising for the knowledge economy and the future of LHSs.

 We posit that a number of policy recommendations and training modernization strategies should be made to foster sustainable embedded research and LHS-related career paths, to continue to attract top talent to programs like the HSI Fellowship, and to raise awareness on the embedded researcher career pathway and the attributes of high-performing LHSs. For example, in Canada, there is a dearth of early career award opportunities for embedded research, and full-time embedded researcher positions are not yet available in abundance. Few organizations other than universities, teaching hospitals and a select number of health authorities are eligible to hold federal research grants, which hampers the ability of embedded researchers to develop programs of research. Additionally, the incentive and reward systems in academia and the broader health system are misaligned, with academia valuing publications over real-world impact,^51^ and the health system valuing relevant, timely, actionable research over publications. Unlike in the academic setting, to fully harness the skills and contributions of PhD graduates in careers within the health system, a shared vision and continued collective action on the part of universities, HSOs and research funders to continue training modernization efforts is needed.

## Acknowledgments

 The authors wish to acknowledge the leadership and contribution of Dr. Adalsteinn (Steini) Brown, Dean, Dalla Lana School of Public Health, University of Toronto; and Dr. Stephen Bornstein, Professor, Memorial University of Newfoundland, who co-chaired the Pan-Canadian Training Modernization Working Group during the study period and who led the development of the Pan-Canadian Training Modernization Strategy and inspired the commitment to track the career pathways of Health System Impact Fellows. The authors also wish to thank the health system, academic and funding partners who share a commitment to embedded research and support the Health System Impact Fellowship program, and the National Cohort Training Program’s leadership and all fellows for their commitment to evidence-informed health system improvement.

## Ethical issues

 The study data were obtained from publicly available sources (social media for career information). The sex data were obtained as per CIHR’s *Applicant Consent Requirements* (section 6.1). Additionally, while sex as a demographic factor was available from self-reporting data and allowed for sex-based analyses in career outcomes (generally not significant), other demographic variables such as race or ethnicity were not available on social media nor in program reporting.

## Competing interests

 Authors declare that they have no competing interests.

## Authors’ contributions

 MM conceptualized the design and methods. BK completed acquisition of data and analysis. BK and MM drafted. ET and RHG contributed to drafting and critical review of the manuscript. RHG provided supervision.

## Funding

 This work was supported by the Canadian Institutes of Health Research’s Institute of Health Services and Policy Research (CIHR-IHSPR).
